# SEI Formation and Lithium-Ion Electrodeposition Dynamics in Lithium Metal Batteries via First-Principles Kinetic Monte Carlo Modeling

**DOI:** 10.1021/acsenergylett.4c02019

**Published:** 2024-10-07

**Authors:** Saul Perez-Beltran, Dacheng Kuai, Perla B. Balbuena

**Affiliations:** † Department of Chemical Engineering, Texas A&M University, College Station, Texas 77843, United States; ‡ Department of Chemistry, Texas A&M University, College Station, Texas 77843, United States; § Department of Materials Science and Engineering, Texas A&M University, College Station, Texas 77843, United States

## Abstract

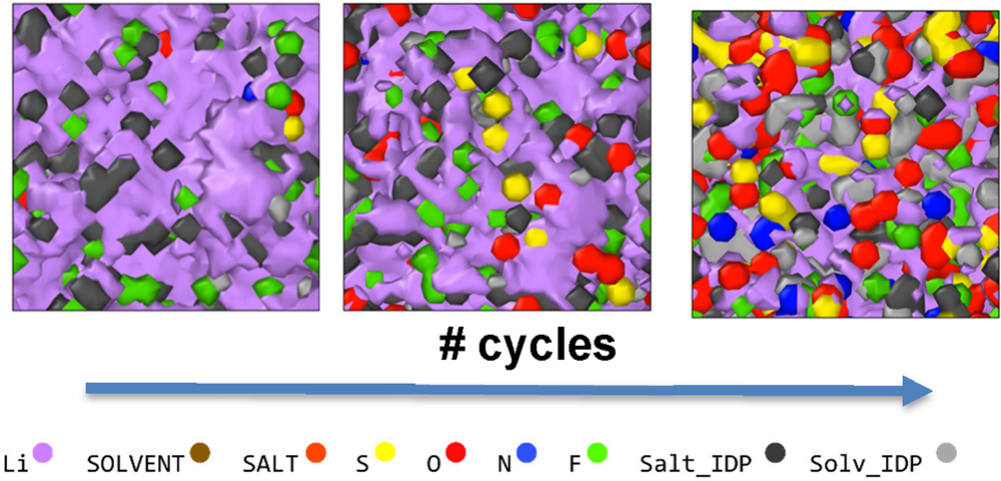

The stabilization and enhanced performance of lithium metal batteries (LMBs) depend on the formation and evolution of the Solid Electrolyte Interphase (SEI) layer as a critical component for regulating the Li metal electrodeposition processes. This study employs a first-principles kinetic Monte Carlo (kMC) model to simulate the SEI formation and Li^+^ electrodeposition processes on a lithium metal anode, integrating both the electrochemical electrolyte reduction reactions and the diffusion events giving place to the SEI aggregation processes during battery charge and discharge processes. The model replicates the competitive interactions between organic and inorganic SEI components, emphasizing the influence of the cycling regime. Results indicate that grain boundaries within the SEI facilitate faster lithium-ion transport compared to crystalline regions, crucial for improving the performance and stability of LMBs. The findings underscore the importance of dynamic SEI modeling for further development of next-generation high-energy-density batteries.

There is an urgent need for further advancements in high-energy density battery technologies to maximize the potential of renewable energies.^1−3^ In this context, lithium metal positions itself as the best material for anode electrodes, as it has the lowest electrochemical potential (−3.04 V vs standard hydrogen electrode) and provides an exceedingly high specific capacity (3.86 Ah/g).^3^ However, the mass commercialization of lithium metal batteries (LMB) faces significant challenges, particularly the stabilization of lithium-ion (Li^+^) electrodeposition and diffusion processes that occur within the passivation layer known as the Solid Electrolyte Interphase (SEI),^4−6^ which inevitably forms at the lithium metal–electrolyte interface due to the instability of current electrolyte formulations at the potentials at which lithium metal anodes operate.^7^

The engineering of the SEI aims for a chemically and mechanically stable, elastic, self-healing, and electronic insulating layer with high Li^+^ conductivity that ensures high Coulombic efficiency.^8^ A typical SEI is formed from a mix of organic and inorganic compounds derived from a self-limited electrolyte decomposition process during the initial cycles of the battery. To achieve this, the current dominant strategy leverages on the lessons learned from Li-ion batteries to come up with innovative electrolyte formulations that help tune the properties of the SEI layer.^9^ This approach has led to the development of high concentration electrolytes (HCEs), localized high concentration electrolytes (LHCEs), and electrolytes with fluorinated solvents, among others.^10−13^ The enhanced salt coordination with Li^+^ ions in HCE electrolytes produces an inorganic-rich anion-derived SEI with suppressed dendrite formation through reduced solvent reactivity and a broader electrochemical stability window,^14^ while LHCEs combine the benefits of HCEs with reduced viscosity and better interfacial stability.^15^ Still, these electrolyte formulations face compatibility issues with high-voltage spinel LNMO electrodes,^16^ encounter safety challenges,^17^ and are potentially expensive due to the higher salt concentrations and/or specialized additives required. On the other hand, solvent fluorination allows for a flame-retardant, single-solvent, low-concentration electrolyte formulation with improved oxidative stability. This promotes the formation of a thermally stable, LiF-enriched SEI layer with high Li^+^ conductivity.^18^ However, the elevated ionic diffusion energy barrier in LiF (0.71 eV) does not correlate with the high Li^+^ conductivity observed in LiF-enriched SEI layers,^19^ which has sparked an ongoing debate on the Li^+^ mobility mechanisms in the SEI. Some authors suggest that grain boundaries between disparate inorganic SEI phases, such as LiF and Li_2_O, play a key role in enhancing Li^+^ transport, rather than diffusion mechanisms within the bulk of the inorganic phases.^19,20^

The experimental work around the SEI properties from these innovative electrolyte formulations is paving the way for the eventual incorporation of LMB batteries into the basket of commercially available energy storage technologies.^21^ However, even though experiments, especially recently developed operando techniques,^22,23^ provide descriptive insights into the chemical composition and morphology of the SEI layer based on performance metrics, challenges remain in identifying and understanding the Li^+^ electrodeposition and diffusion processes across the passivated anode’s outermost layer. For the most part, these processes are only quantifiable through advanced simulation approaches.^19,20^ Here, multiscale modeling plays a critical role. By providing detailed atomic and molecular perspectives, predictive capabilities, and a deeper understanding of complex interactions, multiscale modeling not only addresses the early stage and extended SEI formation and diffusion processes but also enhances cost and time efficiency, guides more informed experimentation, and reduces environmental and safety risks.^24,25^

The set of tools available for modeling the SEI dynamics encompasses a wide range of time and space scales. First-principles methods, such as Density Functional Theory (DFT) and ab initio Molecular Dynamics (AIMD), work in synergy to provide an atomic-level understanding of the fundamental bonding interactions and reactive events involved in the early SEI formation. These methods help elucidate the initial chemical and electrochemical mechanisms, along with their respective energy profiles, in systems comprising a few hundred atoms within a time scale of 10^–10^ seconds.^26−28^ Other methods, such as classical reactive molecular dynamics (ReaxFF) and even coarse-grained molecular dynamics (CGMD), leverage the insights gained from these first-principles approaches to produce less computationally expensive yet accurate larger representations suitable for modeling the early SEI evolution in simulation cells containing up to hundreds of thousands of particles on a microseconds scale.^29−31^ Following the same integrative approach, the mesoscale kinetic Monte Carlo (kMC) technique benefits from these atom-scale methods to follow the SEI dynamics over extended cycling conditions while keeping detailed molecular information. The kMC method is about 10^6^ times less computationally expensive than AIMD and handles processes over time scales ranging from seconds to hours,^32−35^ with simulation cells large enough to address the three-dimensional, dynamic, and heterogeneous SEI structure composed of inorganic and organic phases. Previous works from our group and others have already addressed SEI behavior using a multiscale approach that combines density functional theory and ab initio molecular dynamics calculations with the kMC approach. A work focused on the lithium stripping/plating dynamics on lithium–copper nanoalloys,^33^ uncovered a complex relationship between the nanoalloy composition and the properties of the formed SEI interphase. Meanwhile, other studies examined the SEI formation on a lithium metal surface resulting from the decomposition of a 2 M lithium hexafluorophosphate (LiPF_6_) solution in ethylene carbonate (EC) at open circuit conditions.^34,35^ This modeling effort linked diffusional and kinetic effects to the decomposition mechanisms of LiPF_6_ and EC, respectively, leading to a heterogeneous SEI distribution with an innermost inorganic lithium fluoride (LiF) layer, followed by lithium carbonate, and a porous organic film of lithium dicarbonate exposed to the electrolyte. Another work addressed the formation dynamics and structural trends of the SEI interphase in Li^+^ ion batteries using a first-principles stochastic microkinetic model.^36^ This study demonstrated how the composition and structure of the SEI are significantly influenced by the applied potential and the presence of electron tunneling barriers. It provided a detailed understanding of the competitive reduction mechanisms that drive SEI formation, highlighting the complex interplay between organic and inorganic components in determining the SEI’s properties and their impact on battery performance.

The kMC method relies on a predefined set of elementary electrochemical reactions that encompass the various processes governing SEI evolution.^37^ The comprehensiveness of this reaction set is crucial for accurately modeling the different stages of SEI development. These stages include *the formation stage*, which leads to the deposition of the primary SEI components; *the secondary matrix formation*, that contributes to the growth and densification of the SEI; and *the ultimate failure stage*, characterized by uncontrolled SEI growth. This thickening results in electrolyte depletion, hinders effective and evenly distributed Li^+^ electrodeposition, and ultimately degrades battery performance.^38^

This work discusses the three-dimensional kMC modeling of the Li^+^ electrodeposition dynamics and simultaneous evolution of an SEI layer formed on a lithium metal anode upon reduction of an electrolyte formulation *LiFSI-F5DEE* composed of a 1.2 M lithium bis(fluorosulfonyl)imide (LiFSI) salt dissolved in a fluorinated ether solvent, 1-(2,2-difluoroethoxy)-2-(2,2,2-trifluoroethoxy)ethane (F5DEE).^39^ This solvent belongs to the family of fluorinated-1,2-diethoxyethane (fluorinated-DEE) molecules, developed by Zhenan Bao’s research group at Stanford University under the Battery 500 Consortium.^10,13^ These molecules are notable for their structural tunability provided by the ethyl terminal groups of the parent DDE molecule. This tunability allows for selective iterative fluorination, which endows the solvent with high ionic conductivity and improved oxidative stability due to the remarkable electron-withdrawing capability of the fluorinated groups. This specific system is taken as an example to demonstrate the ability of the kMC method to characterize the complex reaction/transport phenomena taking place at an electrode/electrolyte interface during battery cycling. The analysis provides new insights into the effects of the interfacial events on battery performance.

A crucial result from one of our earlier works^39^ was the elucidation of the reaction network corresponding to the interfacial electrochemistry of the LiFSI-F5DEE electrolyte on the lithium metal surface (Figure 1, reactions labeled a-n and b-m with n = 1 to 8, and m = 1 to 4). The DFT calculations showed that electron transfer from the lithium metal to the electrolyte triggered an initial defluorination of the LiFSI (reaction **a-1**) and F5DEE (reaction **b-1**) molecules near the interface without significant activation energy barriers (E_A,i_, i = LiFSI and F5DEE*)*. For the extended fragmentation of LiFSI, the main reduction path involved the S–N bond breaking, leading to the formation of [O=S=O]^2^^–^ and [O=S(=O)(F)N]^2^^–^ (reaction **a-2**). The further reduction of the remaining LiFSI fragments occurred spontaneously with negligible energy barriers through continuous electron transfer from the lithium metal surface without high-spin state intermediates,^39^ resulting in their full decomposition into single ions (O^2^^–^, S^2^^–^, and F^–^). The breakdown of [O=S(=O)(F)N]^2^^–^ followed reactions **a-3** to **a-5**, while the disintegration of [O=S=O]^2^^–^ occurred through reactions **a-7** and **a-8**. The [S–N]^3^^–^ fragment is the only one with a non-negligible  barrier, but it eventually decomposes into its constituent elements following reaction **a-6**.^40^

**Figure 1:**
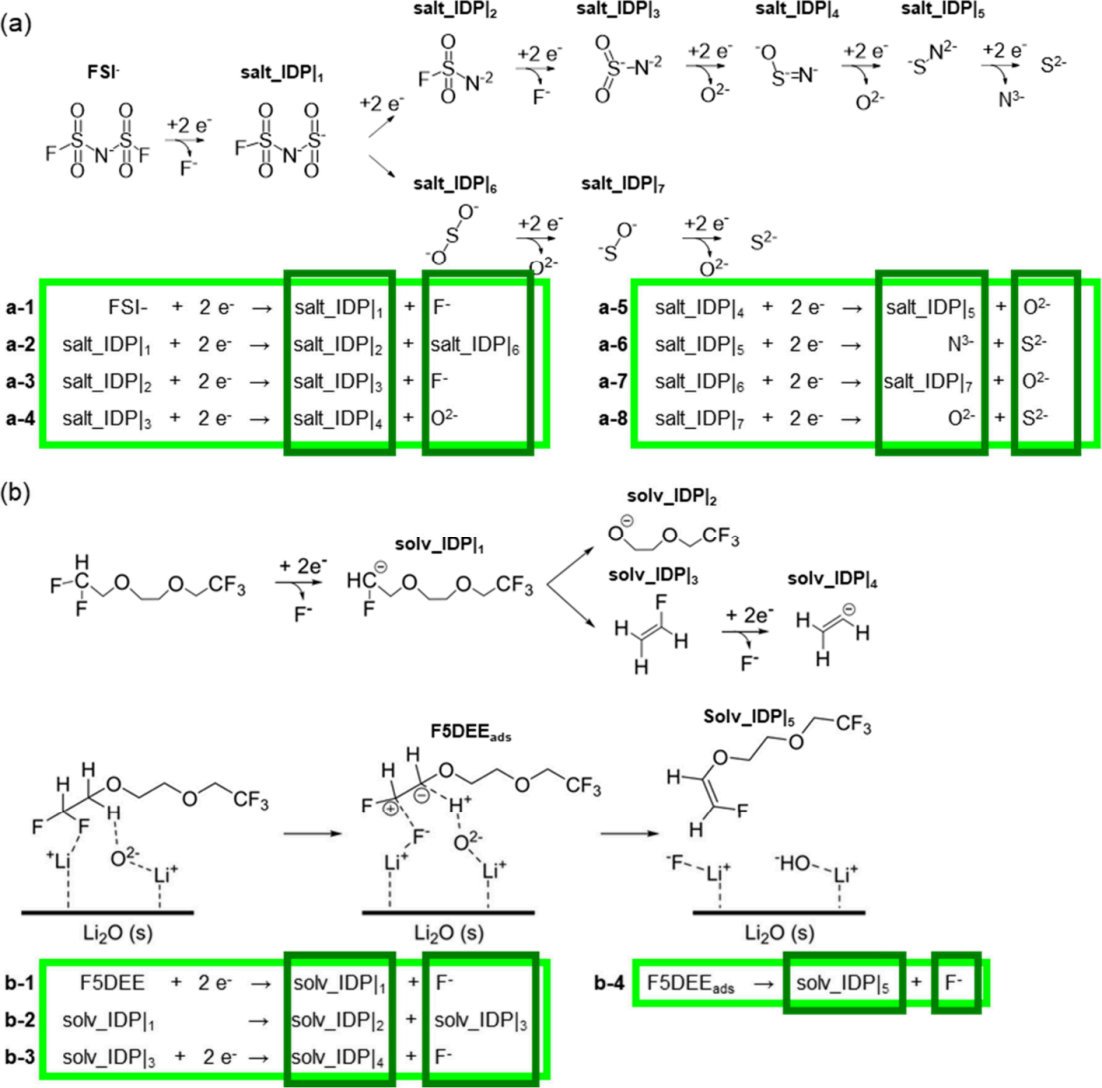
Reaction network path for (a) LISF and (b) F5DEE on lithium metal, highlighting their multiple intermediate decomposition products (salt_IDP and solv_IDP). The figure outlines the set of elementary reactions, enclosed in light-green-colored rectangles, leading to the complete reduction of these two molecules into their constituent species. The dark-green-colored rectangles enclose the intermediate decomposition products and elemental constituents, indicating which particles are subjected to diffusion processes during the growth and densification stage of the SEI, as discussed in the next section.

Similarly, the further reduction of F5DEE (reactions **b-2** and **b-3**) is primarily controlled by electron transfer processes without significant E_A,i_ barriers. This process results in the accumulation of charged vinyl [CH_2_CH]^−^ and [OCH_2_CH_2_OCH_2_CH_3_]^−^ anions on the surface, leading to the formation of an organic amorphous phase. The adsorption of F5DEE on Li_2_O, which is formed upon extended LiFSI decomposition, triggers another decomposition path. This path has a  barrier of 0.364 eV and does not involve an external charge flow, resulting in the formation of LiOH and CF_3_CH_2_OCH_2_CH_2_OCHCH(F). Other works have attributed the presence of LiOH to the native passivation film on lithium metal surfaces, possibly due to water contamination, which then transforms into Li_2_O upon extended cycling.^39,41^ Our modeling strategy in this work uses this reaction network of irreversible elementary reactions to model the electrolyte decomposition without considering the presence of LiOH, assuming it is present only in trace amounts and linking its formation to the presence of impurities and crosstalk reactions with the cathode electrode.^35^

The SEI grows continuously as the electrode cycles due to further electrochemical reactions occurring between the electrolyte and the initial SEI layer, as well as with the electrodeposited Li. The newly formed components from these ongoing reactions aggregate and integrate into the existing SEI structure, thickening and densifying the SEI layer into a mixture of organic and inorganic microphases. Importantly, recent research indicates that the role of interfaces between these microphases is crucial for ion conduction. For instance, the Li^+^ conductivity at the LiF/Li_2_O interface can be as high as 0.59 S/cm at 600 K (1.96 × 10^–4^ S/cm at 300 K), compared to bulk Li_2_O (3.85 × 10^–5^ S/cm at 600 K).^19^

This elevated interfacial ionic conductivity originates from a high carrier concentration, which corresponds to the number of Li^+^ ions available for transport. The carrier concentration follows a Boltzmann distribution related to the carrier formation energy (E_f_), which is the energy required to create a vacancy that can act as a charge carrier; the lower the E_f_ energy, the higher the carrier concentration. The total bonding energy E_B,i_, which depends on the number of nearest sites (n_j_) and the integrated crystal orbital Hamilton population parameter (E_ICOHP,ij_) of the binding atomic pair, is a parameter equivalent to the E_f_ that does not require expensive calculations with large simulation cells,^19^ yet still captures the role of the local environment on the ion mobility. In this work, we introduce the concept of the E_B,i_ dependency on the local coordination environment as a suitable parameter for quantifying ionic mobility during the thickening and consolidation of the SEI layer.

Figure 2 discusses the correlation between the total bonding energy for Li^+^ ions () and their number of bonding interactions. The color map on Figure 2a displays the  energies for the interfacial model of the LiF and Li_2_O phases, with the Li_2_O structure having one Li-terminated facet and one O-terminated facet, both facing Li-terminated LiF surfaces through their (100) surfaces with a low lattice mismatch (∼11%). Earlier calculations demonstrate the thermodynamic stability of this configuration with a binding energy as low as −84 meV/Å^2^.^19,20^ The  energy reaches its lowest values near either interface due to the reduced coordination environment associated with defects and vacancies, while higher values are found in the bulk of the LiF and Li_2_O phases. Figure 2b and Figure 2c confirm the relationship between the Li^+^ local coordination environment and its mobility. The total radial distribution function (RDF) profiles for the Li^+^ bonding interactions, averaged for each of the 17 Li^+^ layers parallel to the LiF|Li_2_O interfaces, show that the L1 and L8 layers, buried in the LiF and Li_2_O bulk phases, have higher coordination peaks relative to the less symmetrical interfacial L6 and L11 layers. This behavior correlates inversely with the mean square displacement (MSD) profiles per layer shown in Figure 2c, where the L6 and L11 layers exhibit steeper slopes compared to the flatter L1 and L8 profiles. The MSD parameter measures the average square distance that Li^+^ ions travel over time and directly relates to their mobility,^42^ with a steeper slope indicating that interfacial Li^+^ ions possess greater mobility.

**Figure 2:**
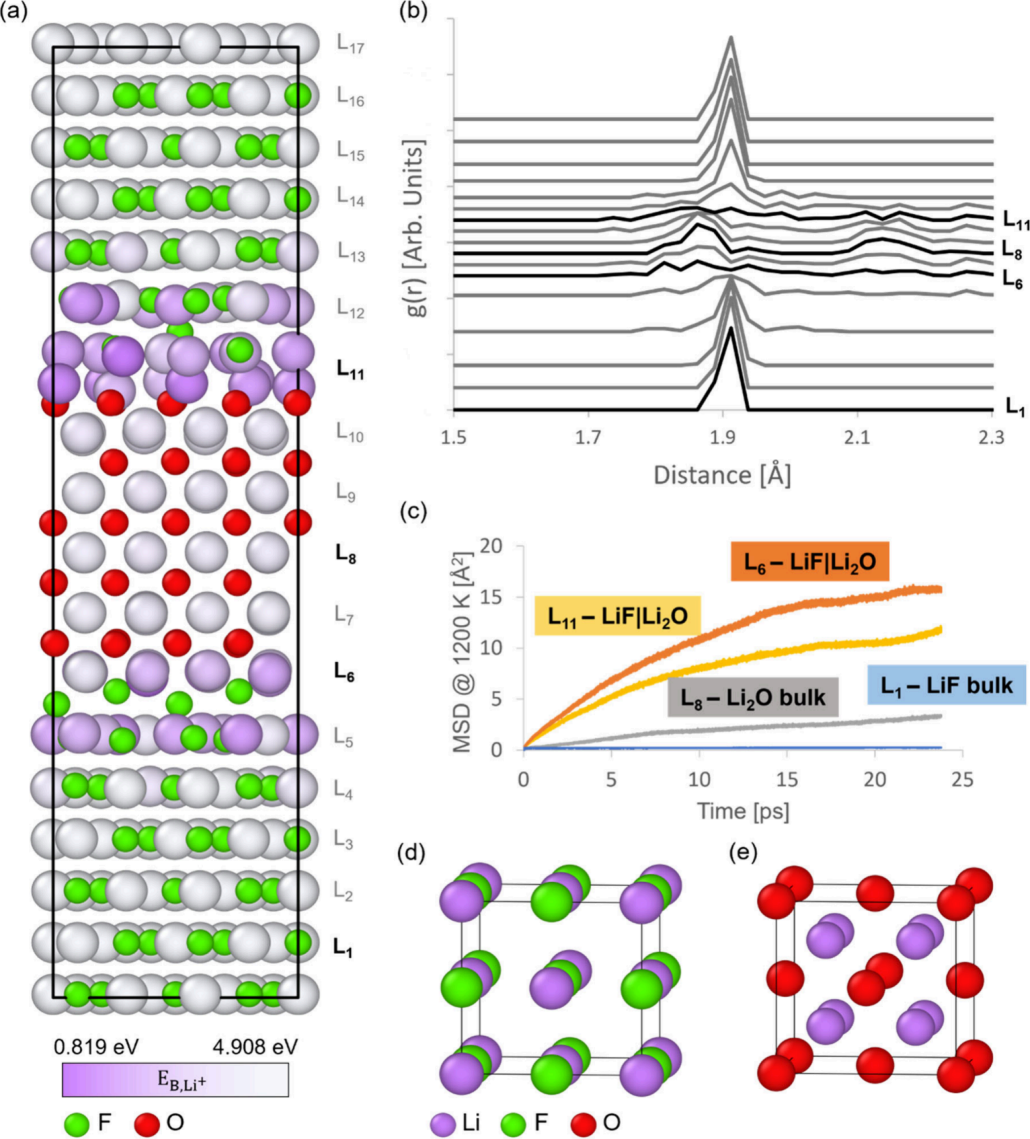
Correlation between  and its corresponding number of bonding interactions. a) Bar color map displays bonding energies at interfaces between Li_2_O and LiF. b) Total radial distribution function profiles for the Li+ bonding interactions. Layers labeled as in a). c) Mean square displacement for Li ions located at the indicated bulk or interfacial layers. d and e) Cell representations for LiF and Li_2_O.

The cell representations for LiF and Li_2_O on Figure 2d and Figure 2e help describe how the kMC approach leverages on variations in the Li^+^ coordination environment to model the role of SEI interfaces in Li^+^ plating/stripping dynamics. LiF crystallizes in a face-centered cubic lattice with a rock salt structure (space group fm3̅m), where Li^+^ ions occupying both corner and face-centered positions bonding with six equivalent F^–^ anions to create LiF_6_ octahedra. In contrast, Li_2_O adopts an antifluoride configuration (space group fm3̅m), with Li^+^ ions residing in tetrahedral positions coordinating with four O^2–^ anions distributed among corner and face-centered sites. This nonequivalent positioning of Li^+^ ions in LiF and Li_2_O induces a substantial plastic deformation occurring along the LiF|Li_2_O interfaces, with Li^+^ ions frequently adopting undercoordinated off-lattice positions, as Figure 2a illustrates, even though both phases share the same space group fm 3̅ m.

Table 1 lists the E_ICOHP,ij_ parameters driving the evolution of the inorganic SEI microphases. By calculating the cumulative energy barrier for anionic mobility (E_B,i_ = n_j_E_ICOHP,ij_), based on the number of atomic pair interactions n_j_, we can understand the factors influencing the Li^+^ and anionic mobility on the surface and within the SEI layer. The set of parameters governing the pair bonding interaction with the intermediate decomposition products (salt_IDP and solv_IDP) are chosen based on performance metrics throughout the simulation to ensure stable SEI growth and coalescence of the anion- and solvent-derived amorphous SEI phases.^34,35^

**Table 1:** Pair Bonding Interaction Parameters Used in the kMC Simulation

Atomic pair	E_ICOHP,ij_ [eV]
Li–Li	0.1037
Li–F	0.7146
F–F	0.0336
Li–O	0.8027
O–O	0.0615
Li–S	0.7439
S–S	0.0735
Li–N	0.9000
N–N	0.1164

The structural framework from which the simulation cell replicates is a unitary cubic box with a lattice parameter a_0,_ and periodic boundary conditions applied in all three spatial directions (Figure 3a). The pair-bonding interaction energy E_ICOHP,ij_ is independent of the magnitude of the a_0_ parameter; therefore, we set a_0_ to 4.47 Å, which is the average value between lithium metal and the main inorganic components present in the SEI (LiF, Li_2_O, Li_2_S, and Li_3_N). This cubic system contains a fixed number of lattice sites arranged in pairs along the body diagonals. The repeating pattern hosts a face-centered cubic (FCC) lattice with its four octahedral and eight tetrahedral interstitial sites. Additionally, this arrangement allows for the conceptual overlay of a body-centered cubic (BCC) lattice within the same spatial framework. This capability enables the replication of the crystalline structures of lithium metal and the inorganic solid electrolyte interphase (SEI) components within our simulation cell, accurately approximating the Li^+^ coordination environment at the interfaces between these SEI components. Figures 3 illustrate the construction of a lithium metal cell with its BCC configuration derived from the BCC sites of the basic cubic box, and the structures of Li_2_O and LiF with their antifluorite and rock salt configurations, respectively, along with the anion and Li^+^ coordination for each case.

**Figure 3:**
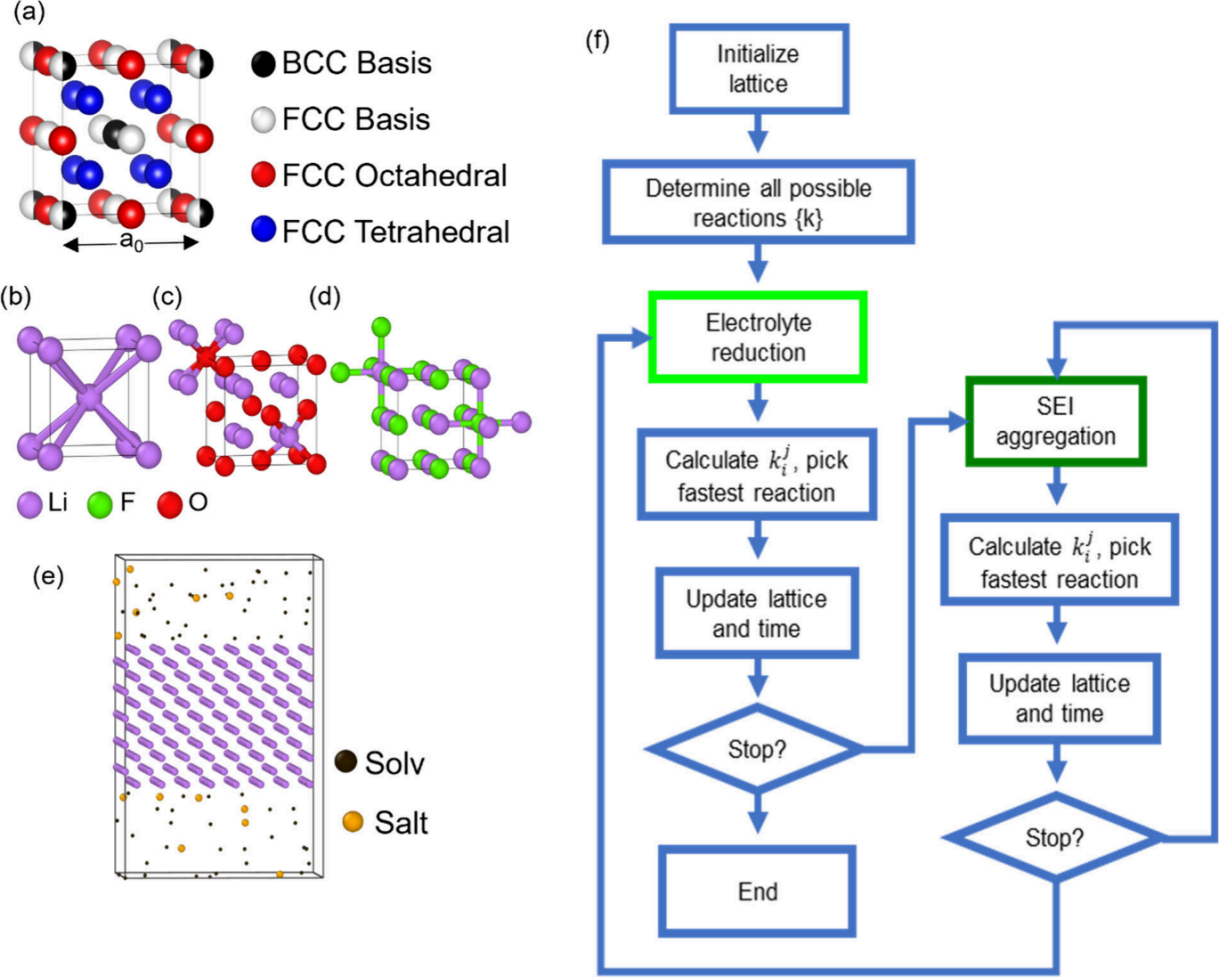
Modeling framework used in our kMC simulations: (a) Unitary cubic box from which the simulation cell replicates, highlighting its lattice sites; the BCC lattice sites overlay the FCC basis sites, including the FCC octahedral and tetrahedral sites. (b) Metallic BCC lithium constructed from the unitary cubic box, along with cubic representations for (c) Li_2_O and (d) LiF, highlighting the anion coordination environment in each case. (e) Generic simulation scenario with a lithium metal slab interacting through its (001) surface with LiFSI and F5DEE electrolyte particles randomly distributed up to their respective molarities, assuming additive volumes. (f) Flow diagram under which the FRM algorithm operates. The light-green loop addresses the electrolyte reduction reactions, while the dark-green loop addresses the exchange reactions driving the SEI aggregation processes.

Figure 3e shows the simulation scenario with a lithium metal slab interacting with a liquid electrolyte through its exposed (001) surfaces. The LiFSI-F5DEE electrolyte consists of a 2 M LiFSI salt fully dissolved in a fluorinated solvent F5DEE (6.5 M), whose particles are randomly distributed throughout the empty space within the simulation cell. Each electrolyte particle occupies a lattice site without accounting for the molecular volume distinctive to each molecule. Hence, packing the solvent molecules to the corresponding molarity, approximated from their density and molecular weight, provides a better approximation of the collision dynamics against the lithium metal surface.

Conversely, Figure 3f outlines the flow diagram under which the first-reaction method (FRM) operates to model the time-dependent electrochemical reaction rates involved in the SEI evolution under discharging and charging regimes. The list of all possible events taking place is split into two simulation loops. The first one corresponds to the deposition of the primary SEI components via the electrolyte reduction paths discussed in Figure 1 (light-green-colored), while the second loop addresses the aggregation and integration of the released SEI components densifying the SEI through the secondary matrix formation stage (dark-green-colored). The diffusion processes driving the SEI aggregation proceed via exchange reactions between neighboring lattice sites. We use the set of parameters listed in Table 1 to compute the energy barrier for ionic mobility (E_B,i_ = n_j_E_ICOHP,ij_) whenever each of these exchange reactions takes place.

The need for two simulation loops arises from the disparate rates at which the electrolyte reduction and the SEI aggregation takes place. The elementary reactions of the electrolyte reduction processes are spontaneous, hence significantly faster than the SEI aggregation and integration processes that depend on local mobility constrains. Tuning the number of iterations of the aggregation loop before another reduction reaction takes place ensures a stable modeling of the SEI growth process.

The rate of reaction (k_i_^j^) for a given i^th^ species undergoing a type of reaction j^th^, including the deposition of primary SEI components through reduction processes and the SEI growth and densification through diffusion reactions, abides by the transition state theory described by an Arrhenius-type expression (eq 1). The parameter ν represents the frequency and orientation of molecular collisions that could lead to the reaction taking place. E_i_^j^ denotes the energy barrier, α_i_^j^ is the transfer coefficient parameter that measures the effective reaction sensitivity to an applied potential (E), E_0,i_^j^ corresponds to a reference potential, k_B_ is the Boltzmann constant, and T is the absolute temperature. In general, this expression for the rate of reaction is valid within a temperature interval where the reaction mechanism remains invariable, and the modeling parameters ν and α_i_^j^ can be fitted to match the SEI deposition rate from experiment accounting for the limited cross-sectional area of the simulation cell. This approach helps compensate for errors in the energy barriers assumed for the aggregation of the anion- and solvent-derived amorphous phase.
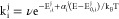
1

The pseudorandom number generator (PRNG) developed by Pierre L’Ecuyer operates the stochastic engine choosing the fastest reaction.^43^ In each iteration of the simulation loops, the PRNG engine generates the random number ξ needed to calculate the time associated with every possible reaction (Δt_i_^j^ = −ln ξ/k_i_^j^, ξ ∈ (0,1)). Hence, the reaction with the smallest Δ*t*_*i*_^*j*^ takes place to update the simulation cell.

Figure 4a shows the tetragonal cell used in our simulation. The horizontal axes are 40a_0_ wide and the vertical ones are 160a_0_ high. The lithium metal is a five layers slab, spans indefinitely along the horizontal axes, and it faces the randomly distributed electrolyte particles on both of its (001) surfaces. Our model does not simulate the electrolyte liquid structure but focuses on the Li^+^ electrochemical plating and stripping processes, the electrolyte reduction reactions, and the subsequent SEI integration and aggregation processes. Figure 4b shows the SEI layer formed after 100 h following a charging–discharging regime with each complete cycle lasting 2 h approximately. The simulation subjects the lithium metal to a regime that mimics the electrolyte reduction and Li^+^ plating in a high-voltage environment (4.4 V), as well as Li^+^ stripping processes during discharge (2.8 V).

**Figure 4:**
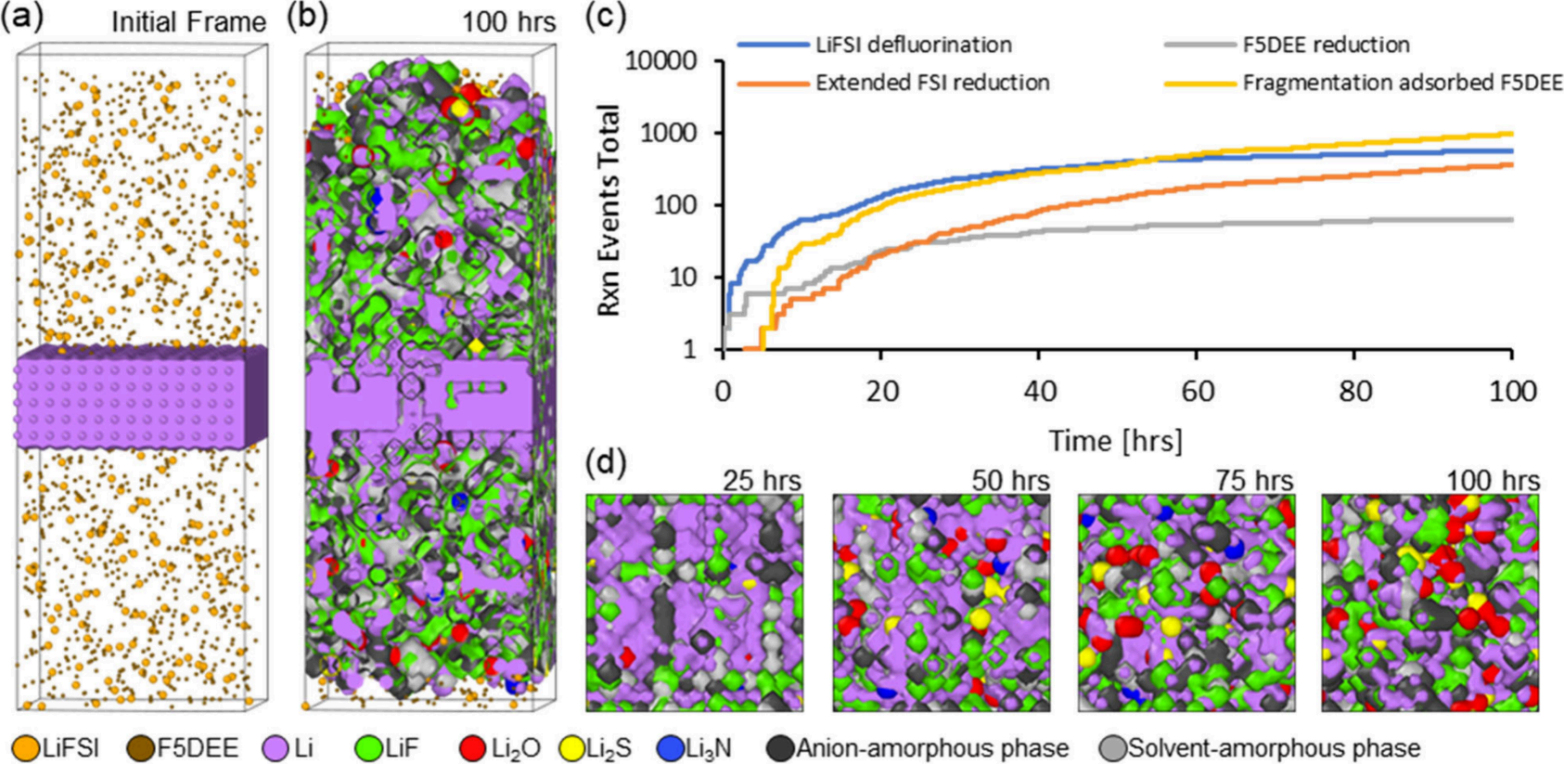
(a) Tetragonal cell used in our simulation with a five-layer lithium metal slab facing randomly distributed electrolyte particles on both (001) surfaces. The model focuses on Li^+^ plating and stripping, electrolyte reduction, and SEI integration processes. (b) Snapshot of the kMC-predicted SEI layer formed after 100 h under a charging–discharging cycling regime. (c) Sequence of reactions showing early SEI formation from LiFSI and F5DEE defluorination, and subsequent contributions of SEI aggregation reactions. (d) Li^+^ electrodeposition dynamics with reduced lithium accumulating at grain boundaries between inorganic SEI phases, highlighting greater Li^+^ mobility at the LiF|Li_2_O interface and the role of grain boundaries in SEI structure and stability.

The SEI aggregation proceeds through diffusion reactions, with multiple inorganic and organic phases building up the heterogeneous SEI layer, as shown across both vertical directions in Figure 4b. The sequence of reactions is also plotted in Figure 4c. The defluorination reactions of LiFSI and F5DEE (reactions a-1 and b-1) drive the early SEI formation, primarily resulting from the coalescence of lime-green-colored LiF particles viewed in Figure 4d, along with charcoal and light-gray-colored phases corresponding to the anion-derived and solvent-derived amorphous phases.

The X-ray diffraction data, combined with pair distribution function (PDF) analysis from our earlier work on the SEI formation on lithium metal from the LiFSI-F5DEE electrolyte,^39^ indicate that the fragments building up these anion- and solvent-derived phases are predominantly made from short sulfur-based S–N/F/O and carbon-based C–O/F/H bonds. The comprehensive characterization of the bonding interactions within these phases falls beyond the scope of the modeling strategy developed in this work, which instead focuses on the Li^+^ electrodeposition dynamics and the SEI morphological evolution over extended cycling.

As the simulation progresses, the extended FSI^–^ reduction (reactions a-1 to a-8) occurs, leading to the formation of inorganic SEI phases (Li_2_O, Li_2_S, and Li_3_N), which thicken the SEI layer while further consuming the active lithium metal particles. In agreement with earlier PDF analysis, the extended F5DEE reduction takes place simultaneously (reactions b-2 and b-3), indicating a competitive decomposition process with LiFSI, in which the ratio between the anion- and solvent-derived amorphous phases varies over time.

The sequence of frames in Figure 4d details the Li^+^ electrodeposition dynamics on the evolving SEI landscape. Notably, the electrochemically active light-purple-colored reduced lithium predominantly accumulates at the grain boundaries formed within the inorganic SEI phases. Our discussion from Figure 2 highlighted greater Li^+^ mobility, where we observed lower binding energies  for undercoordinated Li^+^ ions at the LiF|Li_2_O interface compared to those buried in the bulk phases. Furthermore, the pair bonding interactions listed in Table 1 indicate that Li^+^ pair bonding interactions in inorganic phases (LiF, Li_2_O, Li_2_S, Li_3_N) are approximately 7-fold stronger than the metallic lithium E_ICOHP,LiLi_ bonding energies. This behavior justifies the Li^+^ electrodeposition observed in our simulation, which conforms to the grain boundaries formed between the inorganic and organic domains upon electrolyte decomposition. These results align with observations reported in an earlier work focused on the lithium diffusion mechanism between the three major inorganic SEI components: LiF, Li_2_O, and Li_2_CO_3_,^20^ where it was demonstrated that the grain boundaries within the SEI serve as faster pathways for lithium diffusion compared to the crystalline regions, significantly influencing the overall ion transport and stability of lithium-based batteries.

The surface Li^+^/Li^0^ ratio plotted in Figure 5a (left vertical axis) provide further insights into the overall electrochemical activity of the passivated lithium metal slab in our simulation. The Li^+^/Li^0^ ratio indicates the overall availability of electrochemically active lithium for plating and stripping processes from the total number of lithium particles present close to the electrode surface. Initially, all particles making up the pristine lithium slab surface are reduced to their metallic state (Li^0^) and are readily electrochemically active. However, once the electrolyte reduction reactions take place, the Li^+^/Li^0^ ratio increases because the slab surface undergoes an oxidation process that consumes lithium not only from the slab itself but also from the Li^+^ particles plating from the electrolyte. The SEI evolution pictured in Figure 4d shows a reduction in the covered area of electrochemically active light-purple-colored lithium compared to that of the heterogeneous SEI. This indicates that the effective lithium reduction from plating processes that occur in the high-voltage regime (Li^+^_electrolyte_ → Li^0^_electrode_) becomes less efficient over time. The surface becomes less electrochemically active as it becomes increasingly populated by inactive Li^+^ ions engaged in strong bonding interactions within the inorganic bulk phase, compared to the reduced Li^0^ domains conforming to the SEI grain boundaries.

**Figure 5:**
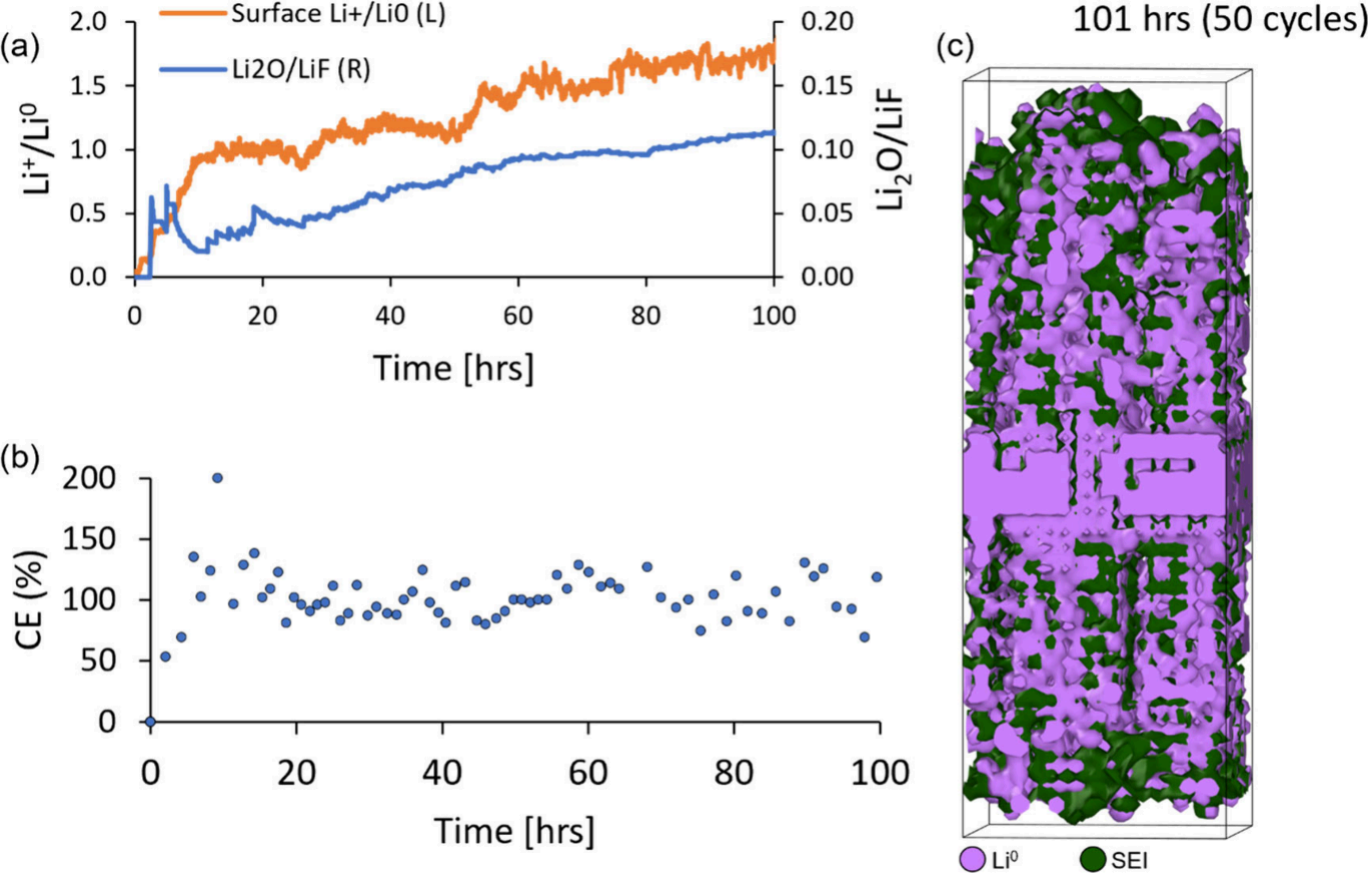
(a) Surface Li^+^/Li^0^ ratio (left axis) and Li_2_O/LiF ratio (right axis) over the course of the simulation. The Li^+^/Li^0^ ratio reflects the availability of electrochemically active lithium, initially high but decreasing due to oxidation and SEI formation. The Li_2_O/LiF ratio starts low, peaking between five to 10 h, indicating dynamic anion reduction and SEI composition changes. (b) Coulombic efficiency (CE%) showing high values during initial cycles due to abundant Li^0^ particles, with a decrease after 25 h as SEI thickens and Li^0^ availability drops. (c) Distribution of Li^0^ particles versus deep green SEI after 100 h, illustrating Li^0^ aggregation near the core and diminishing toward the surface. SEI densification leads to thicker organic and inorganic phases, thinner grain boundaries, and reduced electrode performance.

Figure 5a (right vertical axis) overlaps the Li_2_O/LiF ratio during cycling, providing further details on the anion reduction dynamics and competitive decomposition with the F5DEE solvent. The Li_2_O/LiF ratio starts with very low values during the initial cycles and then peaks between five to 10 h of simulated cycling. This behavior qualitatively agrees with our earlier observations via Rietveld refinement on the SEI evolution from the LiFSI-F5DEE electrolyte on a lithium metal surface, indicating suppressed Li_2_O formation in the initial cycles.^39^ Later, Li_2_O population increases upon further anion reduction, while the LiF growth from simultaneous anion and solvent defluorination processes remains relatively constant throughout the simulation.

This reduction in the electrochemical activity becomes evident with the estimation of the Coulombic efficiency (CE % = e^–^_discharge_/e^–^_charge_ * 100) over the course of the simulation, as plotted in Figure 5b, where e^–^_discharge_ and e^–^_charge_ are the electrons exchanged during discharge and charge reactions, respectively. The CE is based on the number of reaction events at high- and low-voltage regimes and the number of electrons in each elementary reaction outlined in Figure 1 for both the LiFSI and F5DEE decomposition. The CE is predominantly above 100% during the first charging/discharging cycles within the initial 25 h of the simulation because the proportion of reduced Li^0^ particles engaged in metallic bonding interactions and available for stripping reactions is greater than the amount of Li^+^ particles engaged in stronger ionic interactions within the inorganic phases. After 25 h, the surface area covered by the heterogeneous SEI layer overwhelms the reduced Li^0^ particles accumulated at the grain boundaries, hence the proportion of Li^0^ particles available for stripping processes dwindles, and the CE efficiency decreases.

Figure 5c shows the distribution of Li^0^ particles in contrast with the deep green overall SEI formed after 100 h of simulated cycling. This view of the simulation cell confirms the conformal aggregation of reduced Li^0^ particles around the heterogeneous SEI structure. The Li^0^ particles are predominantly close to the core of the passivated surface where the pristine lithium slab was initially located, while the cross-sectional area covered by Li^0^ diminishes closer to the surfaces exposed to the electrolyte particles. As the SEI layer densifies, the multiple organic and inorganic SEI domains grow thicker, the grain boundaries funneling the electrochemically active lithium become thinner, and the electrode surface where Li^0^ particles aggregate and are available for electrochemical plating and stripping processes dwindles, leading to diminished electrode performance.

An important conclusion of this work refers to ionic transport for Li plating and for Li stripping and its relationship to SEI growth. Usually, the SEI phases nucleate separately, and generally inorganic phases have a faster development than the organic ones, but the specific details depend on the electrolyte chemistry.^31^ Depending on the electrolyte, the inorganic phases may appear first, since they mostly originate from faster anion decompositions, although the solvents can also contribute to the oxide phase in later stages. The organic phases are usually much more disperse and they are formed by fragments dissolved in unreacted solvent, or in some cases they could form a semipolymeric structure, and usually they do not form a crystalline phase, although it depends on the specific chemistries. Once the SEI forms, ion transport channels arise naturally between nucleating phases. In the initial SEI stages, the nuclei are mostly of amorphous character, and may allow ionic transport through the nascent nuclei structure. However, as the nuclei become more crystalline over time, such transport through the crystalline phase becomes increasingly difficult, and is dependent on the crystal structure and chemical composition. Much faster is ion transport at the grain boundaries, which may be facilitated or not depending on the chemistry and structure of the exposed facets, as discussed in relation to Figure 2. In addition, changes in voltage and charge rate affect such phenomena.

In preliminary studies, we have varied the current density and applied voltage in a series of tests reported in SI (Figures S1, S2, S3). Figure S1 depicts how Li plating proceeds by deposition of Li atoms between SEI phases, illustrating the importance of such ionic diffusion between grains, along with the extent of amorphous character of the nucleating phases. Figures S2 and S3 explore how the reaction decomposition pathways, and electrodeposition reactions differ between low and high voltage regimes. While Figure S2 focuses on changes in the plating and stripping reactions at each cycle, Figure S3 shows a minimum change in the number of solvent (organic) and salt (inorganic) decomposition reactions. The faster inorganic rates are more affected by cycling than the organic. The plating and stripping reactions are very dependent on the applied voltage, and they also have an important dependence on the cycling states. As cycling progresses, the chances of ion electrodeposition decrease because the surface becomes blocked by the SEI and the electron transport also becomes more difficult, becoming a trigger of the failure mechanism. In addition to Figures S1 to S3, Figure 4 and Figure 5 focus on the changes in surface structure and the effects on Coulombic efficiency that are very relevant for the understanding of the electrochemical cell performance. Additional work based on systematic variations of voltage and charge rate for a given electrolyte on Li deposition morphologies, as well as understanding the differences in transport rates for amorphous and crystalline phases at various interfaces will be discussed in future reports.

One of the goals of this work is to help predicting cell failure. So far, no electrolyte results in a cell performance without failures. However, it may be possible to design chemistries yielding minimum failure and extended lifetimes. Although a thin SEI thickness is not a necessary and sufficient condition to mitigate failure, most of the SEI layers that grow without control exhibit a high electronic conductance,^44^ and a sudden catastrophic behavior. In this work we emphasize that both the SEI compositions, morphology, and properties, and the metal electrodeposition morphology and properties are important. From the observations of the evolution of these phenomena during cycling, one clear conclusion is that the cell dies when plating stops, which may be triggered by electrolyte consumption, SEI completely covering the surface, SEI properties unfavorable to ionic transport and favorable to electronic transport. These are general observations, and more work is needed to outline specific rules that can be related to categories of electrolytes.

Li electrodeposition morphologies have an obvious impact on failure, especially if needle-type plating structures are formed that can grow all the way to the cathode and short-circuit the cell. Mesoscale modeling approaches have been used to get new analyses of the effects of SEI structure and properties and their effects on plating and stripping phenomena. Mukherjee et al.^45^ provided important insights into dendrite growth triggered by the interactions between SEI and Li deposits. They concluded that dendrite growth may be induced by ion depletion at the anode-SEI interfaces due to transport limitations at high reaction rates or low temperatures. Another suggested cause is the existence of spatial variations in reaction kinetics at the anode/electrolyte due to SEI inhomogeneities. This view agrees with the idea of transport limitation that we suggested because of clogging of the grain boundaries. Zhang et al.^46^ evaluated local stresses and deformation in the SEI phase due to Li growth, suggesting tuning SEI stability as a form of ion transport regulation. Again, the idea of SEI stability and ion transport regulation goes along with the findings in this work. Since the method is based on a detailed analysis of the chemistry of the system, the present kMC approach can also provide rules to complement the continuum model discussed by Zhang et al. More recently, Mukherjee et al.,^47^ built on these previous works and introduced the effect of SEI heterogeneities which may induce currents and stress hot spots during metal plating. The net effect is suggested as affecting interfacial stress-contributions to overpotentials that may be significant factors that lead cells into failure. In relation to this point, we expect the current discussion to be insightful feedback to mesoscale models that help facilitating the description of chemo-electro-mechanical behavior of these complex interfaces.

Additional SEI dissolution studies are also needed in this field. Previous work studying SEI dissolution in contact with a DME solvent, for LiF, Li_2_O, Li_2_CO_3_, and LiOH^48^ suggested that their dissolution were not thermodynamically favorable, with LiOH the most favorable of this group to dissolve. However, amorphous SEI nuclei or surface Li ions may perhaps dissolve in certain electrolytes. Interestingly, we recently reported^39^ that crystalline LiOH may become amorphous over cycling. It is possible that the dissolution trend of amorphous LiOH may become favorable. Thus, further analysis and incorporation of these reactions into the kMC model should be of interest.

In summary, this study presents a comprehensive mechanistic model for the SEI formation and the Li^+^ electrochemical plating and stripping reactions on a lithium metal anode in a cycling regime using a first-principles kMC approach. The findings highlight that in the LiFSI-F5DEE electrolyte system, the SEI’s composition and structure are significantly influenced by the aggregation of the SEI components, with organic and inorganic phases competing for dominance. Grain boundaries between disparate inorganic SEI phases, such as LiF and Li_2_O, are identified as faster pathways for lithium-ion transport, enhancing overall SEI conductivity. It confirms the conformal aggregation of the electrochemically active Li^0^ particles around the heterogeneous SEI distribution. Additionally, the efficiency of lithium reduction during plating processes decreases over time due to the densification of the SEI layer and the increasing prevalence of strong ionic interactions within the inorganic bulk phase. The simulated SEI evolution qualitatively agrees with experimental observations, providing a predictive framework for future SEI studies and electrolyte formulation designs. This work underscores the importance of dynamic SEI modeling for the development of next-generation high-energy-density batteries.

## Methods

. The C++ GNU Compiler Collection system (version 11.3.0) and the GNU Scientific Library (version 2.7) power our in-house kMC algorithm, which is tailored for SEI modeling. This algorithm was adapted from an earlier implementation optimized for studying the dealloying of Pt-based nanoparticles for oxygen reduction reaction catalysts.^32^

The Vienna Ab initio Simulation Package (VASP, version 5.4.5) was used to estimate the pair bonding interaction parameters (E_ICOHP,ij_), and model the interfacial LiF|Li_2_O structure using the density functional theory (DFT) method for the structural optimizations and the ab initio molecular dynamics (AIMD) simulations for extended interfacial relaxation processes.^49,50^ The crystallographic data for lithium metal, and the inorganic SEI structures (LiF, Li_2_O, Li_2_S, Li_3_N) was collected from the Materials Project Database.^51^ The generalized gradient approximation based on the Perdew–Burke–Ernzerhof functional (GGA-PBE) was used to describe the electronic exchange and correlation effects.^52^ Conversely, the projected augmented wave formalism was employed to model the electron–ion interactions using a global break condition for the self-consistent iterative loop set to 10^–4^ eV.^53^

The structural optimizations were executed before and after the AIMD calculations using a kinetic energy cutoff set to 520 eV with convergence criteria set to 0.05 eV/Å and a reciprocal Monkhorst–Pack grid of 0.05 Å^–1^. Finally, for the extended AIMD simulations, a temperature of 900 K was maintained using the canonical NVT ensemble. The simulations used a time step set to 1.5 fs, an energy cutoff of 400 eV, and a computationally less expensive gamma-point grid density. The software package Local Orbital Suited Toward Electronic-Structure Reconstruction (LOBSTER, version 5.0.0)^54−59^ was used for the estimation of the pair-bonding interaction parameters (integrated crystal orbital Hamilton population parameter (E_ICOHP,ij_)). The calculation proceeded using Bunge’s local basis functions for each of the atomic species involved with an absolute charge spilling not greater than 3.97% in each case.

## Supporting Information Available

Tests for the effects of voltage and cycling on the reactions considered in this work: Surface morphology evolution (Figure S1), Changes in the number of Li atoms deposited or stripped on the anode surface as a function of voltage and cycle number (Figure S2), and Effect of voltage on main reactions (Figure S3) (PDF)
