# Supplementary material for: SEI Formation and Lithium-Ion Electrodeposition Dynamics in Lithium Metal Batteries via First-Principles Kinetic Monte Carlo Modeling
Source: ACS Energy Lett. 2024 Oct 7;9(11):5268–78. doi: 10.1021/acsenergylett.4c02019 (PMC11555676; doi:10.1021/acsenergylett.4c02019)
Supplement: Supplementary file 1 — nz4c02019_si_001.pdf [file nz4c02019_si_001.pdf]

Supporting Information to:

SEI Formation and Lithium-Ion Electrodeposition Dynamics in Lithium Metal Batteries via First-Principles kinetic Monte Carlo Modeling

Saul Perez-Beltran[1], Dacheng Kuai,[1,2] and Perla B. Balbuena[1,2,3*]

1 Department of Chemical Engineering, Texas A&M University, College Station, TX, USA 77843

2 Department of Chemistry, Texas A&M University, College Station, TX, USA 77843

3 Department of Materials Science and Engineering, Texas A&M University, College Station, TX, USA 77843

* Corresponding author: balbuena@mail.che.tamu.edu

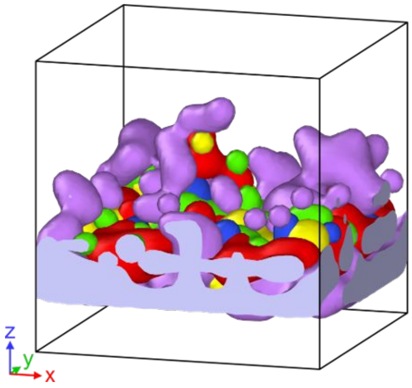

Figure S1. Snapshot of Li deposition morphology during cycling illustrates how Li (purple) deposits in the grain boundaries between SEI domains (red is Li_2O, green is LiF, yellow is LiSx, blue is LiNx. Some interfaces are more favorable than others for Li ions plating or stripping, but as cycling progresses even the less favorable are utilized.

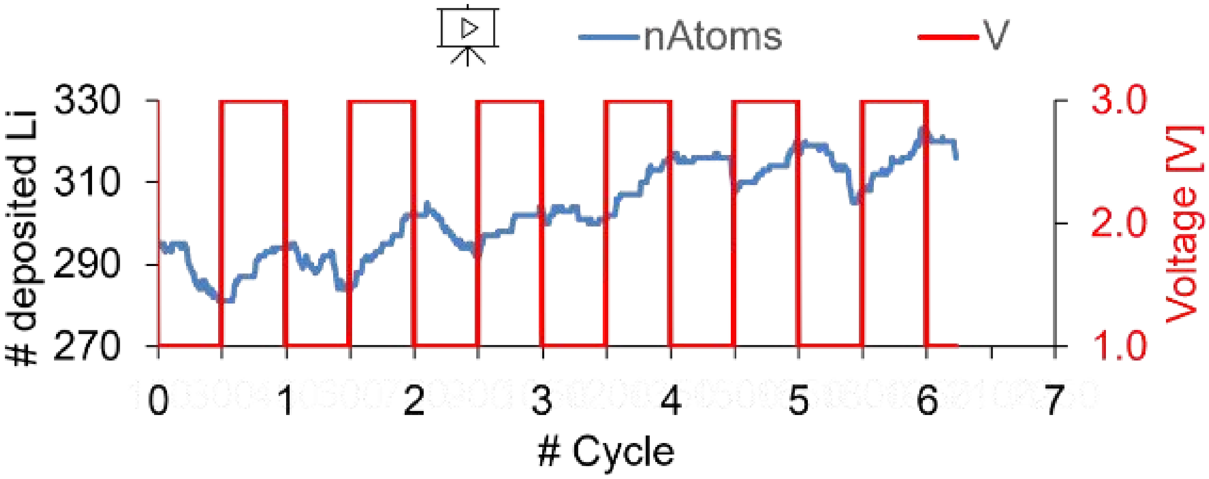

Figure S2. Changes in the number of deposited Li atoms on the anode as a function of voltage. The number decreases during stripping, increases during plating. The difference between them is used to compute the Coulombic efficiency, that varies over cycling.

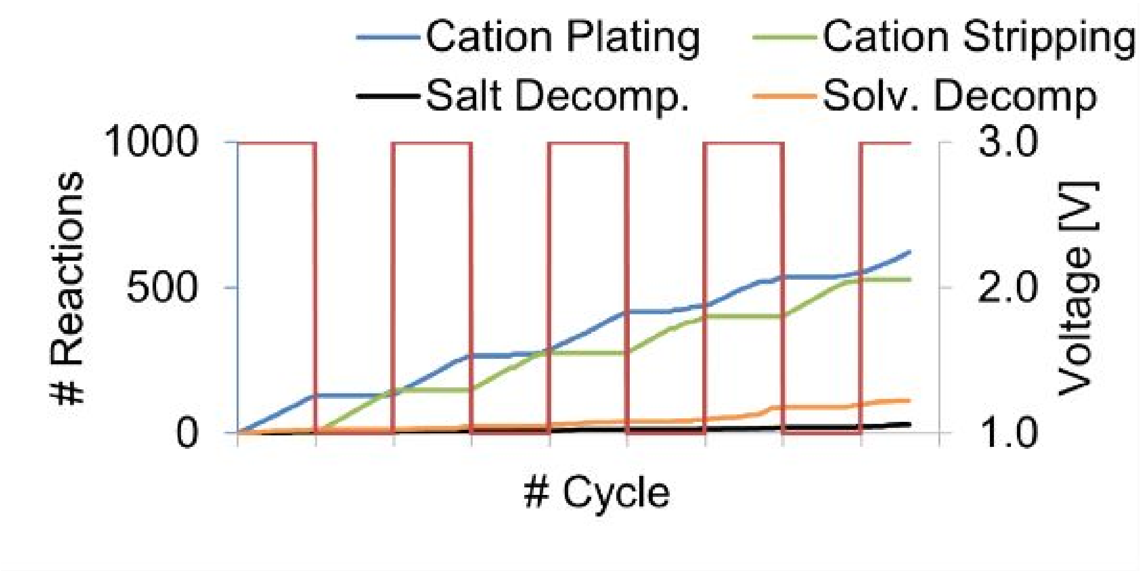

Figure S3: number of reactions of each type as a function of voltage and cycle (charge or discharge): plating (blue), stripping (green), solvent decomposition (orange), salt decomposition (black).

Figure S1 depicts how Li plating proceeds by deposition of Li atoms between SEI phases. This means that such ionic diffusion between grains is very important. Another important property for ion transport is the flexibility (amorphous character) of the nucleating phases.

Figures S2 and S3 addresses the question regarding how the decomposition pathways differ between low and high voltage regimes. While Figure R2 focuses on changes in the plating and stripping reactions at each cycle, Figure R3 shows a minimum change in the number of solvent (organic) and salt (inorganic) decomposition reactions. The inorganic are faster than the organic, but over cycling there are also important changes. The plating and stripping reactions are more affected, and they also have an important dependence on the cycling state. As discussed above, as cycling progresses, the chances of ion electrodeposition decrease because the surface becomes blocked by the SEI and the electron transport also becomes more difficult. This relates to the failure mechanism as discussed in relation to the last comment.

In addition to Figures S1, S2, and S3, Figure 4 in the main text shows the evolution of the various SEI formation reactions and that of the surface during the first 100 hours (50 cycles). The reaction kinetics determines the evolution of each of these reactions and the formation of specific products on the surface. Figure 5 (main text) and associated discussion also focus on

the changes in surface structure and the effects on Coulombic efficiency that are very relevant for the understanding of the electrochemical cell performance.
